# Development of Human ILCs and Impact of Unconventional Cytotoxic Subsets in the Pathophysiology of Inflammatory Diseases and Cancer

**DOI:** 10.3389/fimmu.2022.914266

**Published:** 2022-05-26

**Authors:** Michela Calvi, Clara Di Vito, Alessandro Frigo, Sara Trabanelli, Camilla Jandus, Domenico Mavilio

**Affiliations:** ^1^ Department of Medical Biotechnologies and Translational Medicine (BioMeTra), University of Milan, Milan, Italy; ^2^ Unit of Clinical and Experimental Immunology, IRCCS Humanitas Research Hospital, Milan, Italy; ^3^ Department of Pathology and Immunology, Faculty of Medicine, University of Geneva, Geneva, Switzerland; ^4^ Ludwig Institute for Cancer Research, Lausanne, Switzerland

**Keywords:** innate lymphoid cells (ILCs), natural killer (NK) cells, ILC-poiesis, cytotoxicity, unconventional subsets, inflammation, cancer

## Abstract

Innate lymphoid cells (ILCs) were firstly described by different independent laboratories in 2008 as tissue-resident innate lymphocytes mirroring the phenotype and function of T helper cells. ILCs have been subdivided into three distinct subgroups, ILC1, ILC2 and ILC3, according to their cytokine and transcriptional profiles. Subsequently, also Natural Killer (NK) cells, that are considered the innate counterpart of cytotoxic CD8 T cells, were attributed to ILC1 subfamily, while lymphoid tissue inducer (LTi) cells were attributed to ILC3 subgroup. Starting from their discovery, significant advances have been made in our understanding of ILC impact in the maintenance of tissue homeostasis, in the protection against pathogens and in tumor immune-surveillance. However, there is still much to learn about ILC ontogenesis especially in humans. In this regard, NK cell developmental intermediates which have been well studied and characterized prior to the discovery of helper ILCs, have been used to shape a model of ILC ontogenesis. Herein, we will provide an overview of the current knowledge about NK cells and helper ILC ontogenesis in humans. We will also focus on the newly disclosed circulating ILC subsets with killing properties, namely unconventional CD56^dim^ NK cells and cytotoxic helper ILCs, by discussing their possible role in ILC ontogenesis and their contribution in both physiological and pathological conditions.

## 1 Introduction

Starting from 2008, several independent laboratories around the world identified new players of innate immunity in both humans and mice (1–4). These cells, named innate lymphoid cells (ILCs), are a heterogeneous group of lymphocytes lacking recombination-activating gene (RAG)-dependent rearranged antigen-specific receptors.

ILCs originate from the common lymphoid progenitor (CLP) and require the common γ chain of the interleukin (IL)-2 receptor and the transcriptional repressor ID2 for their development (2–4). They are considered the innate counterpart of adaptive T lymphocytes: ILCs share with T cells the transcription factors governing their differentiation and the same cytokines in response to inflammatory insults (5, 6), which allows the classification of ILCs into different subsets. Indeed, the ILC1, ILC2 and ILC3 subsets produce T helper (Th)1-, Th2- and Th17/22-cytokines, respectively. Furthermore, given their phenotypic, developmental and functional similarities, Natural Killer (NK) cells, the innate counterpart of cytotoxic T lymphocytes, are now grouped together with ILC1s, whereas lymphoid tissue inducer (LTi) cells, belong now to group 3 ILCs (5, 7, 8).

Despite these similarities, ILCs arise from distinct developmental pathways and display unique epigenetic and transcriptional programs with respect to T cells, thus suggesting nonredundant roles of ILCs in immunity (9).

Differently from NK cells, that are mainly circulating lymphocytes, helper ILCs are primarily tissue resident cells and have been found in both lymphoid and non-lymphoid tissues. Indeed, they are particularly enriched at the mucosal surfaces of several organs, such as gut, lungs and skin, where they play a pivotal role in tissue homeostasis and disease, by promoting immune responses, inflammation, tissue repair and tolerance to commensal microbiota (10–12). Despite being a rare population in peripheral blood (PB), several lines of evidence indicate that circulating helper ILCs are characterized by a unique pattern of cytokine receptors, thus suggesting that they are not exclusively tissue resident (9, 13). Moreover, given their innate nature, ILCs represent one of the primary sources of pro- and anti-inflammatory cytokines during the early stages of the immune responses (14–17).

Although progresses have been made in understanding the role of ILCs in the maintenance of tissue homeostasis, in immune-defense and in tumor immune-surveillance, there is still much to learn concerning ILCs, especially in humans.

In this review we will summarize the principal features of ILCs, focusing mainly on circulating subsets. Moreover, we will provide an overview on ILC development in humans. In this context, we will focus on the newly disclosed circulating ILC subsets with cytotoxic properties, namely CD56^dim^CD16^neg^ NK cells and cytotoxic helper ILCs, by discussing their possible role in ILC ontogenesis and their contribution in both physiological and pathological conditions.

## 2 General Features of ILCs

**Figure 1:**
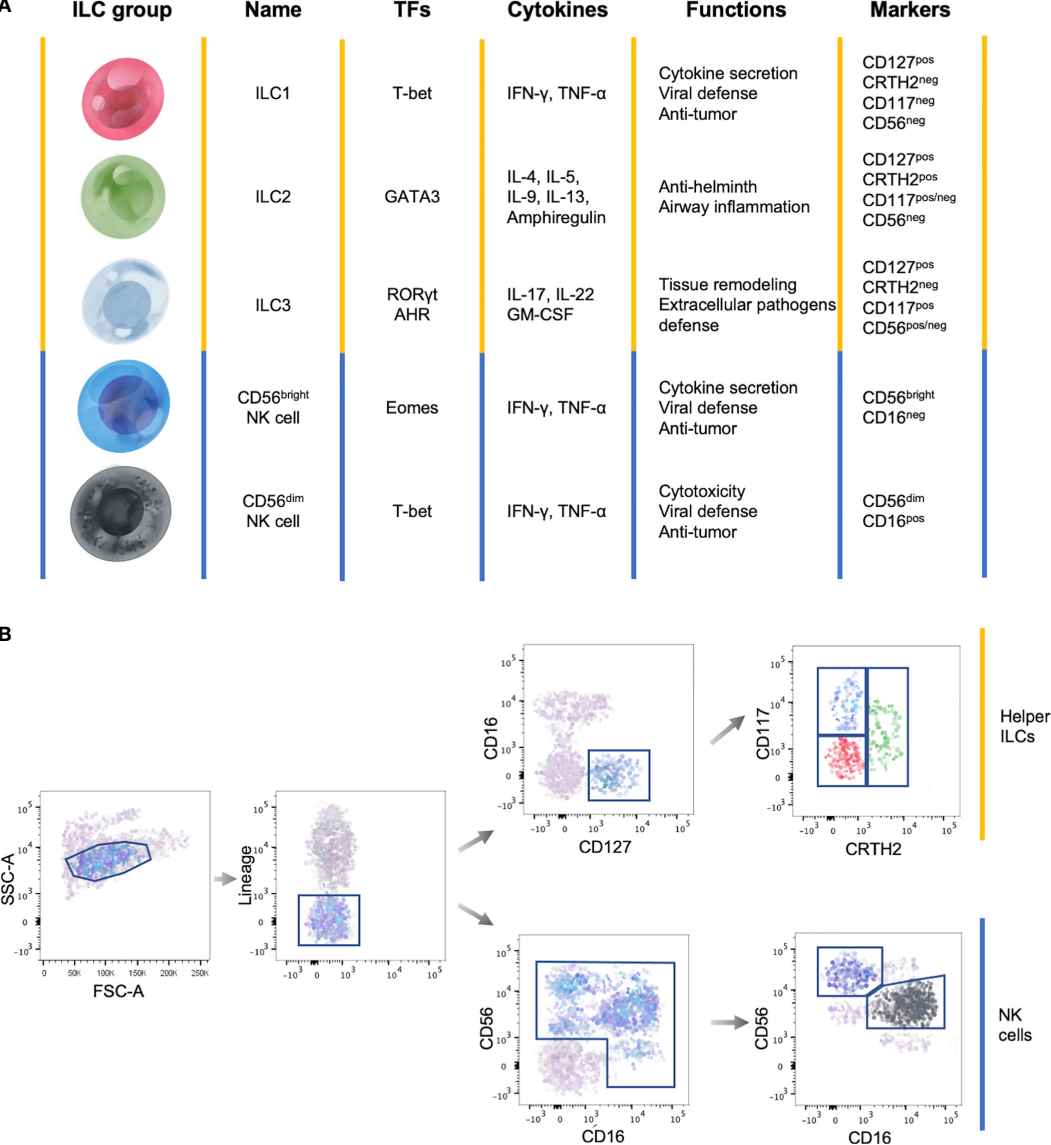
General features of ILC subsets. **(A)** Summary table showing the main transcription factors (TFs) governing the development, the cytokine production, the functions and marker expression in the different ILC subsets. **(B)** Representative flow cytometry gating strategy to identify circulating ILC subsets among Lineage^neg^ lymphocytes. Yellow lines identify helper ILCs, while the blue lines identify NK cells.

ILCs are subdivided into three main groups based on the cytokine production, genetic signature and transcription factors involved in their development (**Figure 1A**).

Total circulating ILCs, which comprises helper ILCs and NK cells, are identified as lineage (CD3, CD14, CD15, CD19, CD20, CD33, CD34, CD203c and FcϵRI) negative lymphocytes (**Figure 1B**) (17). This lineage markers can be used also to identify tissue resident ILCs, with the exception of human tonsil ILC3s which express CD33 (18). Total helper ILCs are defined as CD16^neg^ cells that constitutively express the IL-7 receptor-α chain (CD127) and the different subsets are identified according to the expression of CRTH2, cKit (CD117) and CD56: ILC1s are CRTH2^neg^CD117^neg^CD56^neg^, ILC2s are CRTH2^pos^CD117^pos/neg^CD56^neg^ and ILC3s CRTH2^neg^CD117^pos^CD56^pos/neg^ (**Figure 1B**) (5, 17, 19). However, it has been shown that ILC2s, in some cases, can express CD56 (20). In addition, among lineage negative cells, NK cell subsets are identified according to the differential expression of CD56 and CD16 (**Figure 1B**) (21).

Despite the phenotype of the different ILC subsets is well defined, their precise distribution across organs and species needs to be refined. Indeed, ILC subset distribution and cytokine production profiles are impacted by environmental cues, that enable a prompt response in case of inflammatory insults without the *de novo* recruitment of ILC subsets (22, 23).

### 2.1 Group 1 ILCs

NK cells and ILC1s have been grouped together in group 1 ILCs. Indeed, both subsets are characterized by the production of interferon-γ (IFN-γ) and tumor necrosis factor α (TNF-α) in response to IL-12 and IL-18 (5, 7, 24). Moreover, IL-15 is required for the differentiation, homeostasis, and function of both NK cells and ILC1s (25). Similarly to NK cells, ILC1s express natural cytotoxicity receptors (NCRs) (26) and the T-box transcription factor expressed in T cells (T-bet) (25, 27). In addition, both ILC1s and NK cells require the expression of HOBIT TFs, encoded by *Zfp683*, for their differentiation and functional programs (28–30).

Despite their overlapping features, NK cells and ILC1s are two phenotypically and functionally distinct immune cell subsets. Indeed, while ILC1s require T-bet for their development, NK cells need T-bet expression only for maturation and require the expression of the transcription factor Eomesodermin (Eomes) for differentiation (25, 27). Moreover, ILC1s are fundamentally tissue-resident lymphocytes, whereas NK cells can circulate across lymphoid organs *via* the bloodstream and lymphatic system to act as immune sentinels. Indeed, NK cells are unique in their ability to recognize and kill virally infected and malignantly transformed cells, through the balance of the signaling that NK cells receive from their repertoire of activating and inhibitory NK cell receptors (31, 32).

Circulating NK cells comprise two main subsets, a cytotoxic CD56^dim^ and a regulatory CD56^bright^ NK cell subset. CD56^dim^ NK cell subset accounts for up to the 90% of circulating NK cells. They show high baseline perforin expression and are endowed with cytotoxic abilities against target cells not expressing or downregulating the major histocompatibility complex (MHC) class I molecules on their surface. They preferentially produce cytokines in response to direct target cell interactions rather than *via* monocyte-derived cytokines stimulation (33). On the other hand, CD56^bright^ NK cells represent the 10% of circulating NK cells, while they are enriched in peripheral and lymphoid tissues (34). Differently from CD56^dim^ NK cells, and similarly to ILC1s, CD56^bright^ NK cells are poorly cytotoxic, can rapidly secrete cytokines, including IFN-γ, following stimulation by monocyte-derived cytokines (35).

### 2.2 Group 2 ILCs

Group 2 ILCs are involved in several processes, including lipid metabolism, protection against parasites and accumulate during type 2 inflammation in the airways (36). Among all ILCs, ILC2s express the highest level of the transcription factor GATA3 and are characterized by the production of Th2-associated cytokines, such as IL-4, IL-5, IL-9, IL-13 and amphiregulin (AREG) in response to IL-25, IL-33 and thymic stromal lymphopoietin (TSLP). Hence, they are considered the innate counterpart of Th2 cells (37–39). Of note, several lines of evidence indicate that ILC2s can produce higher levels of cytokine than T cells, thus suggesting their primary role in the innate local immunity against infections in different organs (40). Indeed, they have been described in a variety of human tissues, including tonsils, bone marrow (BM), spleen, skin, adenoids, adipose tissue, lung lymph nodes (LNs) (41).

ILC2s are characterized by the expression of the transcription factor BCL11B which controls their identity (42), the prostaglandin D2 receptor 2 (CRTH2), the IL-33 receptor (IL1RL1 also referred as ST2) and by variable levels of c-Kit (43), all involved in ILC2 localization and function (43–46). More recently, it has been shown that ILC2s are further characterized by the expression of the killer cell lectin-like receptor subfamily G member 1 (KLRG1), a co-inhibitory receptor previously reported in T and NK cells that binds to the members of the cadherin family and is upregulated during infection in response to IL-25 (20, 47).

### 2.3 Group 3 ILCs

Group 3 ILCs, consisting of ILC3s as well as LTis, share features with Th17 cells, including the expression of the transcription factor retinoic acid orphan receptor isoform γt (RORγt), required for their development and function, and the aryl hydrocarbon receptor (AHR) (7, 48). Group 3 ILCs are capable of producing IL-17, IL-22 and granulocyte-macrophage colony-stimulating factor (GM-CSF) in response to IL-23, IL-1β, or natural cytotoxicity receptor ligands (NCR-L), thus mirroring Th17 response (5, 49). Regardless of the functional association with ILC3s, LTis are considered a separate ILC lineage (49, 50). Indeed, LTis are involved in the secondary lymphoid organ formation during embryogenesis and adulthood and in their restoration following infection (51), whereas ILC3s mainly contribute to the immune responses against specific extracellular pathogens and in the maintenance of tissue homeostasis at mucosal sites, where they are mainly localized (48).

ILC3s can be further subdivided according to the expression of the NCR NKp44: human NKp44^pos^ ILC3s, largely co-expressing NKp46, are the majority in adult tonsil and intestine and represent an exclusive source of IL-22, while NKp44^neg^ ILC3s are the major population in fetal LNs and preferentially express IL-17 transcripts (48).

Differently from tissues, ILC3s are under-represented in the circulating lymphocyte pool. However, recently, a subpopulation of cells phenotypically resembling to NKp44^neg^ ILC3s has been described in cord and adult PB. However, most peripheral-blood CD127^pos^CD117^pos^ ILCs express low levels of RORγt, do not produce any of the ILC cytokine signatures following stimulation with IL-1β and IL-23 and their transcriptome is different from that of mature ILC3s present in secondary lymphoid organs (52, 53). Circulating CD127^pos^CD117^pos^ ILCs are instead multi-potent ILC precursors (ILCPs) that retain the ability to give rise to functionally mature helper ILC subsets, as well as to Eomes^pos^ NK cells, after *in vitro* culture with appropriate cytokine mix or after transfer *in vivo* into immunodeficient mice (49, 53).

## 3 ILC Development

ILC-poiesis has been a topic of ardent research in the last several years. Although many issues remain to be disclosed, including the transcriptional regulators that dictate the choice of mature ILC subset fate, the ILC differentiation in mouse has been deeply investigated, thanks to genetically modified rodent models. On the contrary, the study of the human ILC differentiation is still in its infancy because of different reasons. Firstly, the lack of comparable genetic and tracing tools that are available in animal models of lymphoid development. Secondly, the heterogeneity of the studies conducted in terms of definition of progenitor, identification of lineages based solely on cytokine secretion or on few surrogate markers.

**Figure 2:**
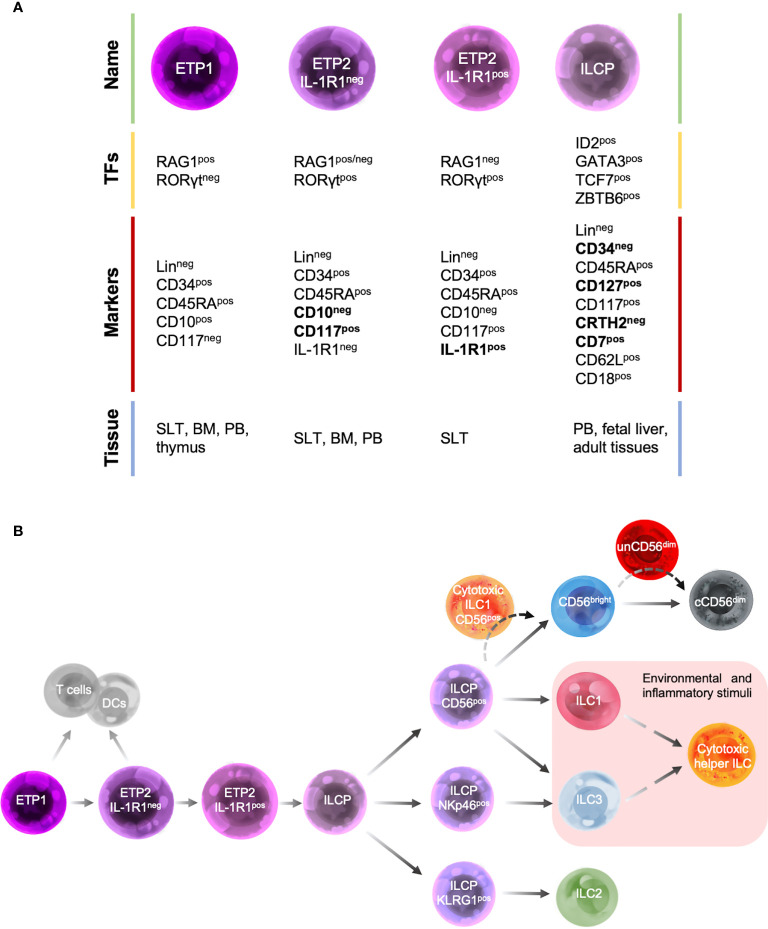
Human ILC developmental stages. **(A)** Summary table of the main transcription factors (TFs), marker expression and tissue localization of common precursors/progenitors. **(B)** Schematic representation of different stages of human ILC-poiesis starting from the early tonsillar progenitors (ETPs), that still retain the ability to give rise to T cells and dendritic cells (DCs), to ILC precursor (ILCPs) that branches into progenitors with restricted differentiation potential and give rise to different mature ILC subsets. Dashed lines indicate the hypothetic developmental pathways of unconventional cytotoxic ILC subsets.

Despite these limitations, over the past years a map of human ILC development has been under construction (**Figure 2**) by taking advantage of both murine ILC-developmental model and the pre-existing model of human NK cell development, based on the identification and characterization of human NK cell developmental intermediates (NKDIs) prior to the discovery of helper ILCs (54).

### 3.1 Human NK Cell Development

Since their discovery in 1970s, NKDIs have been well characterized in both mice and humans as well as the site for NK cell development.

Human NK cells were originally thought to develop strictly within the BM (33, 55). Indeed, CD34^pos^CD45RA^pos^ precursors with *ex vivo* potential for NK cell differentiation were firstly identified in the BM, and then were also found in PB and in extramedullary tissues, including thymus, secondary lymphoid tissues (SLTs), liver, and uterus, at steady state conditions (56–58). Noteworthy, IL-15-responsive CD34^pos^CD45RA^pos^ precursors comprise a relatively higher proportion of total CD34^pos^ progenitor cells (5-10%) in the blood compared to bone marrow (<1%), and in SLT they comprise the major subset of CD34^pos^ progenitor cells (>90%) (59, 60). In this regard, several lines of evidence demonstrated that CD34^pos^CD45RA^pos^ precursors originate in the BM and traffic to extramedullary tissues where later stages of NK cell differentiations can take place, giving rise to tissue-specific and functionally distinct mature NK cell subsets. In particular, tonsils, spleen, and lymph nodes are considered those SLTs hosting the main extra-medullary sites of NK cell development and maturation (57, 59–62).

Originally, five main sequential stages of NK cell maturation were identified: NK cell progenitors (NKPs, stage 1), pre-NK cells (stage 2), immature NK cells (iNK, stage 3) (63–65) and the mature CD56^bright^ (stage 4) and CD56^dim^ (stage 5) NK cell subsets [reviewed in (62)].

Briefly, NKPs and pre-NK cells still express CD34 and retain the ability to differentiate into T cells, dendritic cells (DCs) and other ILCs. Subsequently, the expression of CD122 (IL-2Rβ), together with the downregulation of CD34, marks the irreversible fate decision into NK cell lineage. The commitment of NKPs towards pre-NK cells also required the acquisition of CD117 expression (64, 66). Recently, the stage 2 has been further subdivided into two additional stages: the IL-1R1^neg^ stage 2a, mainly enriched in SLTs and PB, that still retains the ability to give rise to T cells and DCs, and the IL-1R1^pos^ stage 2b, with a commitment restricted to the generation of ILCs, including NK cells (62, 67). IL-1R1^pos^ stage 2b give rise to iNKs whose features, including AHR, CD127, RORγt, IL-1R1, and IL-22 expression, mirror those of Group 3 ILCs (5, 68, 69). Indeed, it is not yet clear if iNKs and ILC3s are entirely overlapping at least in their phenotypic characteristics and further investigation is needed.

The final transition of iNK into mature NK cell subsets is marked by the appearance of CD56 expression and the main functional properties, including cytokine secretion (i.e. TNF, IL-8, GM-CSF, CXCL12 and IL-13, together with IL-22), IFN-γ release and then cytolytic activity (58, 70–72). Two distinct stages 4 CD56^bright^ NK cells have been described: the NKp80^neg^ stage 4a cells, that, despite the bright expression of CD56, are more similar to iNKs cells due to their higher expression of transcription factors RORγt and AHR, their higher production of IL-22 and their preferential localization in SLTs, the NKp80^pos^ stage 4b cells, expressing higher levels of T-bet and Eomes and producing IFN-γ (73). Subsequently, through the acquisition of CD16, Killer Immunoglobulin-like receptors (KIRs) and cytotoxic granules, the fully mature CD56^dim^ NK cells, endowed with cytolytic potential and able to perform antibody-dependent cell-mediated cytotoxicity (ADCC) are generated.

Despite the process of NK cell-poiesis is well defined, with the identification and characterization of other subtypes of ILCs, the old model of NK cell ontogenesis needs to be reassessed and refined in the context of new data about helper ILCs (74).

### 3.2 Murine ILC Development

Murine ILC differentiation is regulated by a wide range of transcription factors, including *Id2*, *Nfil3*, *Zbtb16*, *Tcf7*, *Gata3*, *Ets1*, and *Tox* [reviewed in (75)].

Two distinct progenitors downstream of the murine CLP have been identified, each with restricted ILC potential. These include the CXCR6^pos^α_4_β_7_^pos^CD127^pos^ α-lymphoid precursor (αLP) and the Lin^neg^Thy1.2^neg^CD127^neg^α_4_β_7_^pos^ early innate lymphoid progenitor (EILCP) that along with the other downstream progenitors are most prevalent in murine BM (54, 76). The transcription factor *Nfil3* is required for the generation of αLP. Indeed, *Nfil3* knock out mouse models lack mature ILCs, including NK cells (76–78).

On the other hand, EILCPs, requires the transcription factor *Tcf7* (79). Given the drastic reduction of αLPs together with EILCPs in *Tcf7*-deficient mice, it has been also proposed that αLP could constitute an intermediate stage of maturation between CLP and EILCP (76).

Two additional EILCP subsets with different commitment potential have been identified: the early-stage EILCPs (EILP1s), that can give rise to DCs as well as cytotoxic and helper ILCs, and the committed EILPs (cEILCP) expressing TCF-1 and losing the ability to differentiate in DCs (80).

The EILCP can further differentiate in *Id2*-dependent Lin^neg^Id2^pos^IL-7R^pos^IL-2Rα^neg^α4β7^pos^ common helper-like ILC progenitors (CHILPs), which can give rise to helper-like ILCs (ILC1s, ILC2s, and ILC3s) and LTis but not to NK cells (79, 81) or in *Id2*-independent NK1.1^neg^IL-2Rβ^pos^ NKPs (82, 83).

CHILPs can be separated into two different subsets based on the expression of *Zbtb16*. The Zbtb16^neg^ common ILC precursor (CILCP) which can no longer produce LTi cells (83, 84) and the *Zbtb16^pos^* ILC precursor (ILCP) capable of potentially giving rise to conventional NK cells, ILC1s and ILC2s, but with a reduced ability to differentiate into ILC3s (83–86).

### 3.3 Human ILC Development

According to NK cell developmental model, the most immature ILC progenitors identified in humans are mainly localized in SLTs and were originally described as stage 1 and stage 2 NKIDs and are now defined as early tonsillar progenitors (ETPs) in the context of ILC-poiesis (**Figure 2A**). In particular, ETPs are subdivided into Lin^neg^CD34^pos^CD10^pos^CD117^neg^ ETP1 and Lin^neg^CD34^pos^CD10^neg^CD117^pos^ ETP2 (54, 63). Both ETP1s and ETP2s are multipotent and could also give rise to T cells and DCs *in vitro* (54, 63). ETP2s are heterogeneous in terms of IL-1R1 expression. IL-1R1^neg^ ETP2s have a residual T-cell and DC potential, whereas IL-1R1^pos^ ETP2s are ILC restricted. These population are also characterized by a unique transcription factor signature: ETP1 are RAG1^pos^ and express low levels of ID2 and RORγt, IL-1R1^neg^ ETP2s are ID2^pos^RORγt^pos^ and retain a low expression of RAG1, whereas IL-1R1^pos^ ETP2s are ID2^pos^RORγt^pos^RAG1^neg^ (**Figures 2A, B**) (67). Hence, IL-1R1^pos^ETP2s are the earliest committed human CILCP identified to date.

In addition, a Lin^neg^CD34^neg^CD7^pos^CD127^pos^CD117^pos^CRTH2^neg^ ILC progenitor (ILCP), with phenotypic features that overlap those of stage 3 NKIDs endowed with a restricted potential for ILC generation, has been recently identified in human cord and adult PB as well as fetal liver and several adult tissues. Upon *in vitro* culture with an appropriate cytokine environment or after transfer *in vivo* to immunodeficient mice, these human ILCPs demonstrate their potential for generating all mature helper- and cytotoxic-ILCs (53).

Consistent with their differentiation potential, ILCPs express high levels of transcription factors that have been shown to be essential for mouse ILC development, such as ID2, GATA3, TCF-7 and ZBTB16 (**Figure 2A**). In contrast, moderate to low levels of the lineage-determining transcription factors RORγt, T-bet, Eomes, cytokine receptors (including IL-1R1), and signature cytokines have been found (53). ILCPs show a migratory profile including the expression of L-selectin (CD62L) and β2-integrin (CD18), which would allow these cells to populate the mucosal tissues where they terminally differentiate into mature ILCs in response to local and environmental triggers, during both homeostatic and inflammatory conditions (54, 87, 88). Consistent with this idea, ILCPs were found at mucosal sites where they mature (53).

In light of this evidence, ILCPs might be the equivalents of naïve CD4^pos^ T cells. Indeed, they both express CD45RA and CD62L and the development of ILC subsets from ILCP parallels that of CD4^pos^ T cell subsets from naive CD4^pos^ T cells, with similar polarizing cytokines and transcription factors being required for their differentiation, although the differentiation of naïve CD4^pos^ T cell subsets also depends on TCR signaling and CD28 co-stimulation (22).

Different ILCP populations with a restricted differentiation potential have been recently described and can be identified based on the expression of CD56, NKp46 and KLRG1. CD56^pos^ ILCPs show a restricted potential for NK cells, ILC1s and ILC3s, NKp46^pos^ ILCP predominantly differentiate into ILC3s, whereas KLRG1^pos^ ILC precursors mostly develop into ILC2s (**Figure 2B**) (88, 89).

Adding complexity to this scenario there is the so called ILC plasticity phenomenon. Indeed, it has been demonstrated in both humans and mice, that ILC subsets can switch into another subset depending on the presence of cytokines and NOTCH ligands in their environment. This process is regulated by a complex network of transcription factors. Briefly, ILC2s and ILC3s transdifferentiate into ILC1s in response to IL-1β and IL-12, whereas IL-1β and IL-23 can drive the plasticity of ILC1s and ILC2s towards ILC3s. Despite ILC2s lack the expression of IL-23 receptor, IL-1 β is known to induce ILC2s responsiveness to IL-23 by STAT3 phosphorylation (90). The transdifferentiation of ILC2s into ILC1s or ILC3s can be reversed by IL-4. ILC2s requires TGF-β in addition to IL-1β and IL-23 to differentiate into ILC3s (22). Moreover, NK cells, in a TGF-β-rich tumor environment, transdifferentiate into ILC1-like cells devoid of cytotoxic activity (91, 92). Likely, the plasticity of ILCs is the mechanism of tissue-resident ILCs to dynamically adapt to a given stimulus, such as an inflamed state (23).

## 4 Killer ILC Subsets

Until recently, NK cells were considered the only cytotoxic innate lymphocytes, being functionally associated with CD8 T cells (7). However, owing to the highly plastic nature of ILCs, increasing evidence shows that beside their ability to transdifferentiate between helper subpopulations, these cells can also acquire cytotoxic capacities upon defined environmental conditions (93). Indeed, it has been recently reported that, upon exposure to cytokine cocktails, ILC3s or ILC1s, isolated from human tonsils, secondary lymphoid organs (e.g., spleen) and intestinal tissues of humanized mice, give rise to cytotoxic lymphocytes resembling stage 4a NK cells (94, 95). In particular, hallmark NK cell genes, such as *NCAM-1*, *KLRD1, KLRC1, CD2, CD226* have been reported to be significantly upregulated in ILC3s or ILC1s exposed to IL-12/IL-15, and to be paralleled by the acquisition of cell surface expression of at least some of these markers (e.g. NKG2A, NKG2C, CD2). Moreover, these cells show a weak IFN-γ secretion in response to K562, but an efficient perforin- and granzyme-dependent cytotoxicity, primarily mediated by the Eomes^pos^T-bet^pos^ fraction of stimulated ILC3s. Nevertheless, these cytotoxic responses remain weaker and of slower kinetics as compared to that of conventional NK cells.

Phenotypically, the distinction between human cytotoxic helper ILCs and NK cells is complicated by the fact that many markers are shared, including CD56, CD161, NKp44 and NKp46. The same stands for mouse ILCs and NK cells that share the expression of NKp46 and NK1.1. Possible discriminators to distinguish cytotoxic helper ILCs, or helper ILCs in general, from NK cells include CD127, which is constitutively expressed by both human and murine helper ILC subsets but not by terminally-differentiated CD56^dim^ NK cells (67) and only at intermediate levels on human peripheral blood CD56^bright^ NK cells (96). Noteworthy, in mice, ILCs with cytotoxic potential were also described within NK1.1^pos^CD49a^pos^ cells, lacking CD127 expression (26).

Another marker enabling the discrimination between helper ILCs and NK cells might be the inhibitory receptor CD200R1. The expression of CD200R1 has been described to be specific for ILCs in human blood and tonsils (20), and in mice (84, 97). However, intestinal NK cells have been recently described to express CD200R1, although at lower levels as compared to conventional ILCs (95). Inversely, CD200R1 is poorly expressed by intraepithelial ILC1s in the small intestine, lamina propria and visceral adipose tissue (98). Overall, these results highlight the difficulty to identify a universal marker enabling ultimate discrimination between helper ILCs, including cytotoxic ones, and NK cells. Nevertheless, despite potential overlapping phenotypes and functions, the distinct anatomic distribution of cytotoxic helper ILCs and NK cells argues for complementary roles in the protection against infections or emerging malignancies.

Evidence of *in vivo* ability of helper ILCs to acquire cytotoxic features has been recently shown in different districts, as detailed in the next paragraphs.

### 4.1 Unconventional CD56^dim^ NK Cells

In the context of NK cell development, an additional NK cell subset has been recently identified. This subset, named unconventional NK cells (unCD56^dim^), displays a CD56^dim^CD16^neg^/CD56^low^CD16^low^ phenotype. Barely detectable in the PB, this NK cell subset is mainly enriched in the BM (99, 100). On the other hand, their presence in extramedullary tissues has not been investigated so far.

UnCD56^dim^ NK cell subset expresses surface markers of mature NK cells, such as NKG2D and NKp30 (99). Moreover, this NK cell subset is equipped with lytic molecules, thus suggesting its putative role in mediating cytotoxic responses.

However, their phenotype and transcriptional profile suggest that unCD56^dim^ NK cells are a *bona fide* NK cell subset distinct from activated CD56^dim^ that underwent CD16 shedding mediated by the metalloproteinase-17 (101). Indeed, compared to CD56^dim^ NK cells, unCD56^dim^ NK cells show higher levels of CD27, whose expression was described to decline during NK cell development in mice, and lower levels of markers of terminally differentiated and licensed NK cells, namely CD57 and KIRs, which are acquired at late stages of NK cell differentiation (100). Moreover, unCD56^dim^ NK cells display higher levels of CD25, CD122 and CD127, the receptor chains for IL-2, IL-15 and IL-7 cytokines, which play a major role in controlling NK cell development, homeostasis, survival and activation (100). The chemokine receptor expression pattern of unCD56^dim^ is consistent with a less differentiated phenotype compared to CD56^dim^ NK cells. Indeed, they are characterized by undetectable *CX3CR1* expression, which is usually acquired during NK cell development, and by a higher expression of *CXCR4* compared to the other conventional NK cell subsets, in line with their preferential BM localization (100).

In the context of the lymphopenic environment of patients affected by hematologic malignancies and which underwent haploidentical hematopoietic stem cell transplantation (haplo-HSCT), others and we reported that unCD56^dim^ NK cells are by far the largest subset of NK cells immune-reconstituting in the first 2-4 weeks after the transplant, compensating the low frequency of the conventional cytotoxic CD56^dim^ NK cells (99, 102). These data, together with the transcriptional and phenotypic characteristics of unCD56^dim^ NK cells, intermediate between that of CD56^bright^ and CD56^dim^ NK cells, suggest that this subset could represent an additional or alternative stage of NK cell differentiation that drives the NK cell maturation process (**Figure 2B**) (99, 100).

*In vitro* experimental evidence also suggests that unCD56^dim^ NK cells possess multifunctional ability and superior effector-functions. Indeed, despite poorly present under homeostatic conditions in the PB, they are endowed with potent cytotoxicity, significantly higher than that of CD56^dim^, and an IFN-γ producing capability comparable to that of CD56^bright^ in response to cytokine stimulation (99, 100, 103). On the contrary, immune-reconstituting unCD56^dim^ NK cells, highly expanded early after haplo-HSCT, are anergic due to a high expression of CD94/NKG2A, an inhibitory receptor involved in NK cell differentiation and education, thus further supporting the assumption of unCD56^dim^ NK cells as a distinct NK cell subset and highlighting their key role in NK cell development. Moreover, this observation allowed us to develop a phase II clinical trial (ONC-2020-001) by using an anti-NKG2A humanized monoclonal antibody (humZ270 mAb, IPH2201, monalizumab, AstraZeneca) to block this inhibitory checkpoint, unleashing alloreactive unCD56^dim^ NK cells, thus potentially improving the clinical outcome of haplo-HSCT early after transplant (99, 104, 105).

### 4.2 Cytotoxic Helper ILCs

#### 4.2.1 Tonsillar Cytotoxic ILCs

The *ex vivo* analysis of human tonsil ILCs has shown the existence of a population of CD94^pos^ cells, that co-expressed CD200R1, while negative for CD16, NKp80 and KIRs. In terms of transcriptomic profiles, these cells cluster close to NKp44^pos^ ILC3s, sharing with them the expression of *RORγt*, but being distinct in terms of cytotoxic gene expression (e.g., *Eomes*, *Granzymes*, *Granulysin*) (106). This gene expression pattern has been also correlated with direct cytotoxic activity against the target cell line K562, which is predominant for the CD94^pos^NKp44^neg^ ILC3 subpopulation. Additional evidence of the *in vivo* generation of cytotoxic helper ILCs in human inflamed tonsils has been recently reported by combining bulk and single cell RNA sequencing (scRNAseq), flow- and mass-cytometry studies (107). In particular, it has been demonstrated that ILC3s and ILC1s reside at the terminal ends of a differentiation spectrum. Between these two extremes, four ILC3-ILC1 intermediates, with a NKp44^pos^CD56^pos^ phenotype, emerge by RNA velocity analyses. These intermediates are characterized by the gradual acquisition of genes expressed in conventional NK cells, such as *KLRD1*, *KLRC2*, *GZMB* and *GZMK*, loss of *RORG*, *CD200R1*, *KIT*, *IL7R* and upregulation of *Tbx21* and *IKZF3*.

#### 4.2.2 Circulating Cytotoxic ILCs

We recently identified a CD56-expressing subset of circulating ILCs with high cytotoxic potential that belong to conventional ILC1s, being characterized by the lack of lineage markers, the expression of CD127 and the absence of CD117 and CRTH2 (108). RNA-sequencing analysis revealed a transcriptional profile closer to ILCPs and NK cells rather than ILC1s. These CD56^pos^ILC1-like cells showed distinct nutrient uptake and mitochondrial activity in comparison to helper ILCs and NK cells, low expression of NKp46 and the capacity to produce IL-8, beside IFN-γ, when stimulated with IL-12, IL-15 and IL-18. Through comparison with NK developmental intermediates in terms of phenotype (73), by verifying their presence in patients with severe combined immunodeficiency, in human fetal tissues and during immune reconstitution in humanized mice, and by assessing their capacity to differentiate into conventional ILCs/NKs when cultured on OP9 mouse stromal cells, we concluded that they are related to the developmental stage 4a of NK cells. The cytotoxic machinery of CD56^pos^ILC1-like ILCs comprised DNAM-1, NKp30, NKp80 and TRAIL, as well as the ability to produce perforin, granzyme A, B, K, M and granulysin. Functionally, CD56^pos^ILC1-like ILCs can kill both the MHC-I^neg^ K562 cell line and MHC-I^pos^ cell lines, such as BJAB and U937, in accordance with the absence of KIRs. Their cytotoxic capacity is dependent on the expression of NKp30, NKp80 and TRAIL, since the addition of specific blocking antibodies can inhibit their killing ability. We further investigated their presence and function in acute myeloid leukemia (AML), a hematologic malignancy characterized by a dysfunctional helper ILC compartment (109, 110). The data obtained demonstrated that in AML patients at diagnosis, the cytotoxic machinery of CD56^pos^ILC1-like ILCs is completely impaired, resulting in their inability to kill target cells, either K562 or autologous blasts. On the other hand, AML patients that achieved remission showed a completely restored function of CD56^pos^ILC1-like cells. Interestingly, CD56^pos^ILC1-like ILCs but not conventional NK cells from AML patients at diagnosis had a reduced expression of TRAIL, NKp80 and granulysin, thus suggesting that despite their relatedness they are distinct populations able to differentially react to the microenvironment. Further studies are needed to determine whether this population is an intermediate between helper ILCs and NK cells, or a specific cytotoxic circulating helper ILC subset.

#### 4.2.3 Intestinal Cytotoxic ILCs

In a recent study aimed at creating a high-dimensional tissue map of human NK cells across multiple tissues, cytotoxic helper ILCs have been also identified (111). Indeed, CD56^pos^CD3^neg^ cells expressing CD127, CD56 and CD161 have been found to be enriched in the intestine, as well as in lung-draining lymph nodes and mesenteric lymph nodes. These cells secreted IFN-γ upon stimulation and upregulated CD107a when co-cultured with MHC-I^neg^ targets, suggesting that they might represent either immature NK cells or tissue-resident cytotoxic helper ILC3s, present at selected anatomical sites. These cells might be key in maintaining intestinal homeostasis by cytokine secretion. In that regard, intraepithelial cytotoxic ILCs, very much resembling NK cells, have also been identified by others in the intestinal mucosa (26). These cells are characterized by the expression of CD56, Eomes, T-bet, variable levels of NKp44 and CD103, low levels of CD94 and CD127. This phenotype has not been observed in other anatomic locations, suggesting a selective localization of this subset in the mucosal epithelium of the gut. *In vitro* characterization of these cells revealed that they are able to respond to IL-12 and IL-15 stimulation by secreting IFN-γ, to produce Granzyme B and to express CD107a when exposed to cell targets. Studies in mice showed that these intraepithelial cytotoxic ILCs rely on *Nfil3*- and *Tbx21*-transcription factors, while IL-15 is dispensable for their development, suggesting that, at least in mice, they are distinct from conventional NK cells. The investigation of their putative role in gut inflammation revealed that their frequency is increased in patients with Crohn’s disease as well as in *in vivo* models of colitis, arguing for a direct involvement in pathology. In a more recent study, amplified CD94^pos^CD127^pos^ ILCs has been reported in the intestinal lamina propria in adults, but not in the epithelium, as opposed to the previous study (95). scRNAseq analysis of healthy and Chron’s disease patients’ tissue specimens revealed the existence of two clusters of lamina propria CD94^pos^CD127^pos^ cells, with cytotoxic attributes. These cells express Eomes and CD200R1, but lacked CD16 expression. Heterogeneity was observed in terms of CD161, CD117, CD18, cytotoxic molecules (e.g., granzymes and perforin) and granulysin expression. Specifically, the granulysin^high^ perforin^high^ subpopulation is highly amplified in Chron’s disease patients, arguing for its induction during the inflammatory process. This hypothesis is supported by the observation that CD94^pos^CD127^pos^ ILCs are absent in fetal intestine, where instead NK cells are abundant. The activation of these CD94^pos^CD127^pos^ ILC1-like ILCs in patients with bowel inflammation might have opposite effects: sustain inflammation on the one hand and fulfill bactericidal activities on the other hand. However, additional experimental investigations are needed to confirm this hypothesis. Given the localization of ILC3s at mucosal barriers, these *in vitro* observations argue for a potential role of cytokine-induced NK-cell like ILC3s in providing cytotoxic protection at mucosal sites, where NK cells are low abundant. If and how this NK cell-like activity of ILC3s could be exploited in vaccination settings against viruses or cancer remains to be studied (94).

#### 4.2.4 Liver Cytotoxic ILCs

A very recent work, investigating the immune phenotype of ILCs in hepatocellular carcinoma by scRNAseq, has demonstrated the existence of a cluster of liver-resident ILC1s endowed with cytotoxic potential (112). This cluster is characterized by the expression of cytolytic effector genes, including *FGFBP2*, *FCGR3A*, *CX3CR1*, *GZMB*, *GZMH*, and *PRF1*. Moreover, these cytotoxic ILC1s are mainly enriched in non-tumor tissues, while in tumor samples ILC1s are characterized by higher levels of exhaustion markers, such as *LAG3*, thus suggesting that ILC1s could undergo functional conversion during liver cancer progression. Further, a high accessibility to the granzyme C gene locus and high *GrzmC* transcripts were recently observed in ILC1s purified from murine liver and salivary glands. Granzyme C^pos^ ILC1 could be differentiated from ILC precursors, are ontogenetically distinct from NK cells and do not convert into ILC2 or ILC3. Granzyme C expression is dependent on T-bet, while sustained TGF-β signaling is required for the maintenance of granzyme C^pos^ ILC1 in the salivary gland, but not in the liver. Using the PyMT breast carcinoma model, the authors show that these cells expand and contribute to tumor anti-tumor functions in a TGF-β-dependent manner. If a similar ILC1 subset exists in humans remains to be tested (113).

Overall, cytotoxic helper ILCs have been so far identified within the ILC1 and/or the ILC3/ILCP subsets, but not within ILC2 compartment (**Figure 2B**). However, CD56^pos^ (89), CD94^pos^ (20) and NKG2D^pos^ (114) ILC2s have been reported in different settings, either *in vitro* upon cytokine exposure or *in vivo* in human peripheral blood. It remains to be verified if these cells have direct cytotoxic functions, like their cytotoxic ILC1 and ILC3 counterparts.

## 5 Concluding Remarks

Since their discovery, many efforts have been done to characterize the origin, the function and identity of different ILC subsets. The knowledge regarding ILC biology is continuing to expand and includes the identification and characterization of progenitors, the refinement of mature ILC identities as well as the definition of additional ILC subsets. However, it is of utmost importance to understand if these novel ILC subsets coincide to additional developmental intermediate stages or they are the result of ILC plasticity to adapt to environmental stimuli. Furthermore, emerging evidence highlights the existence of circulating and tissue-resident helper ILCs endowed with cytotoxic potential. These cells, with a phenotype resembling to ILC1s and/or ILC3s/Ps but not ILC2s, most likely are a consequence of environmental and/or inflammatory triggers and could provide early innate defenses against different pathogens, particularly in mucosal tissues, where NK cells are underrepresented.

In the present review, we gave an overview of the current knowledge of ILC biology, mainly focusing on their developmental process. We further summarized the possible developmental pathways of the unconventional cytotoxic ILC subsets recently identified (**Figure 2B**).

Nevertheless, further studies are also needed to deeper characterize the pathways of human ILC development and to understand the differences and similarities with murine ILC-poiesis. In this context, studying the immune-reconstitution of ILC subsets after HSCT certainly represents an important strategy to shedding light on the *in vivo* ILC developmental trajectories at least in periphery. Moreover, the understanding of ILC-poiesis and homeostatic mechanisms driving donor-derived ILC immune-reconstitution as well as determining the acquisition of cytotoxic features, could be of clinical utility. Indeed, it will allow the development of protocols to ameliorate the HSCT outcome based either on adoptive ILC transfer therapies of *ex vivo* generated alloreactive ILCs or on systemic cytokine infusion/blocking antibodies to boost *in vivo* ILC effector-functions. Moreover, given the important role of helper ILCs in tissue immune-surveillance, these novel therapeutic options will find application in the management of solid as well as hematologic cancers and of inflammatory disorders.

## Author Contributions

MC, CDV, AF, ST, CJ, and DM wrote and critically reviewed the manuscript. AF and MC draw the figures. All authors gave the final approval to the manuscript.

## Funding

This work was supported by Associazione Italiana per la Ricerca sul Cancro (IG 2018-21567 to DM), Intramural Research Funding of Istituto Clinico Humanitas (to DM), the Italian Ministry of Health (Bando Ricerca Finalizzata PE-2016-02363915 to DM), SNSF PRIMA fellowship (PR900P3_17972729 to CJ), the Swiss Cancer Research Foundation (KFS 5250-02-2021 to CJ) and the Geneva Cancer League (GCL, 2007 to CJ). MC is a recipient of the Leonelli AIRC fellowship (26580). MC and AF are recipients of competitive fellowships awarded from the PhD program of Experimental Medicine from University of Milan. ST is recipient of a Dr Henri Dubois-Ferrière Dinu Lipatti Foundation research fellowship. We also thank the financial support from Fondazione Romeo ed Enrica Invernizzi.

## Conflict of Interest

The authors declare that the research was conducted in the absence of any commercial or financial relationships that could be construed as a potential conflict of interest.

## Publisher’s Note

All claims expressed in this article are solely those of the authors and do not necessarily represent those of their affiliated organizations, or those of the publisher, the editors and the reviewers. Any product that may be evaluated in this article, or claim that may be made by its manufacturer, is not guaranteed or endorsed by the publisher.
